# Case report: The success of empagliflozin therapy for glycogen storage disease type 1b

**DOI:** 10.3389/fendo.2024.1365700

**Published:** 2024-06-11

**Authors:** Ana Klinc, Urh Groselj, Matej Mlinaric, Matjaz Homan, Gasper Markelj, Ajda Mezek Novak, Andreja Sirca Campa, Jaka Sikonja, Tadej Battelino, Mojca Zerjav Tansek, Ana Drole Torkar

**Affiliations:** ^1^ Faculty of Medicine, University of Ljubljana, Ljubljana, Slovenia; ^2^ Department of Endocrinology, Diabetes, and Metabolic Diseases, University Children’s Hospital, University Medical Centre Ljubljana, Ljubljana, Slovenia; ^3^ Department of Gastroenterology, University Children’s Hospital, University Medical Centre Ljubljana, Ljubljana, Slovenia; ^4^ Department of Allergology, Rheumatology and Clinical Immunology, University Children’s Hospital Ljubljana, Ljubljana, Slovenia; ^5^ Department of Endocrinology, Diabetes and Metabolic Disease, Division of Internal Medicine, University Medical Centre Ljubljana, Ljubljana, Slovenia

**Keywords:** glycogen storage disease type 1B, GSD-1b, empagliflozin, SGLT2 inhibitor, neutropenia, inflammatory bowel disease, case series

## Abstract

**Introduction:**

Glycogen storage disease type 1b (GSD-1b) is characterized by neutropenia and neutrophil dysfunction generated by the accumulation of 1,5-anhydroglucitol-6-phosphate in neutrophils. Sodium-glucose co-transporter 2 inhibitors, such as empagliflozin, facilitate the removal of this toxic metabolite and ameliorate neutropenia-related symptoms, including severe infections and inflammatory bowel disease (IBD). Our case series presents the treatment of three pediatric GSD-1b patients with empagliflozin over a follow-up of three years; the most extended reported follow-up period to date.

**Cases description:**

A retrospective analysis of empagliflozin treatment of three pediatric GSD-1b patients (two male and one female; ages at treatment initiation: 4.5, 2.5 and 6 years) was performed. Clinical and laboratory data from a symmetrical period of up to three years before and after the therapy introduction was reported. Data on the clinical course of the treatment, IBD activity, the need for antibiotic treatment and hospitalizations, neutrophil count and function, and markers of inflammation were assessed. Prior the introduction of empagliflozin, patients had recurrent oral mucosa lesions and infections, abdominal pain, and anemia. During empagliflozin treatment, the resolution of aphthous stomatitis, termination of abdominal pain, reduced frequency and severity of infections, anemia resolution, increased appetite, and improved wound healing was observed in all patients, as well as an increased body mass index in two of them. In a patient with IBD, long-term deep remission was confirmed. An increased and stabilized neutrophil count and an improved neutrophil function enabled the discontinuation of G-CSF treatment in all patients. A trend of decreasing inflammation markers was detected.

**Conclusions:**

During the three-year follow-up period, empagliflozin treatment significantly improved clinical symptoms and increased the neutrophil count and function, suggesting that targeted metabolic treatment could improve the immune function in GSD-1b patients.

## 1 Introduction

Glycogen storage disease type 1b (GSD-1b) is an autosomal recessive disorder of carbohydrate metabolism caused by a pathogenic variant in the *SLC37A4* gene encoding glucose-6-phosphate translocase (G6PT). The role of G6PT is to transport glucose-6-phosphate from the cytoplasm to the endoplasmic reticulum, where it undergoes hydrolysis (1). The impaired G6PT function leads to disturbances in the maintenance of energy and glucose homeostasis (2).

GSD-1b is characterized by neutropenia and neutrophil dysfunction, presumably caused by the accumulation of 1,5-anhydroglucitol-6-phosphate (1,5-AG6P) in the cytoplasm of neutrophils. This metabolite is produced by the phosphorylation of 1,5-anhydroglucitol, a food-derived polyol, which undergoes dephosphorylation in the endoplasmic reticulum, facilitated by transport through functional G6PT (3). Therefore, G6PT dysfunction increases the concentration of 1,5-AG6P in the cytoplasm, leading to an energy deficit in the cell and subsequently weakening respiratory burst and chemotaxis (2, 3). GSD-1b patients have recurrent infections and a higher incidence of inflammatory bowel disease (IBD) and mucosal lesions (4). Until recently, neutropenia-related symptoms were managed with granulocyte-colony stimulating factor (G-CSF) (1). However, with the advances of knowledge in the pathogenesis of neutropenia in GSD-1b, sodium-glucose co-transporter 2 (SGLT2) inhibitors, such as empagliflozin, were found to lower 1,5-AG6P concentration in neutrophils by indirectly inhibiting renal reabsorption of 1,5-anhydroglucitol (3, 5–10). Multiple case reports and multicenter retrospective studies have demonstrated safety and improved clinical outcomes with empagliflozin; however, the published studies mainly encompassed a follow-up period of a few months to two years (6–18).

This article aims to present the experience with empagliflozin treatment in three pediatric patients with GSD-1b over three years, making it the longest-reported follow-up to date.

## 2 Cases description

The Slovenian registry of GSD-1b patients managed at University Children’s Hospital Ljubljana currently comprises three patients: two boys and one girl. We will report the efficiency and safety of empagliflozin treatment in our patients. The course of the treatment will be evaluated based on changes in clinical and laboratory parameters, presented in **Table 1**, over a symmetrical period of up to three years before and after treatment initiation. Prior to empagliflozin introduction, the diagnosis of GSD-1b was confirmed by molecular analysis of the *SLC37A4* gene, which revealed homozygous c.[1042_1043delCT];[1042_1043delCT] variant in Patient 1 (P1), compound heterozygous state c.[1109_1110delCT];[c.81T>A] genotype in Patient 2 (P2), and compound heterozygous state c.[547T>C];[530_531delTG] variants in Patient 3 (P3), with the latter variant being categorized as likely pathogenic.

**Table 1:** Clinical observations and laboratory data before and after empagliflozin administration.

	Sex (M/F)	Current age (years)	Follow-up (months)	Average dose of empagliflozin (mg/kg/day)
Patient 1 Patient 2 Patient 3	M M F	7.5 5 8.5	36 34 31	0.38 0.48 0.57
Equal time frames	**BEFORE TREATMENT**		**AFTER TREATMENT**	
NEUTROPHYL DYSFUNCTION				
	Absolute neutrophil count—median, range (/µL)			
Patient 1 Patient 2 Patient 3	1200 (300–2500) 1260 (0–2300) 550 (100–970)		1750 (800–4100) 1850 (1120–3370) 1600 (1200–3100)	
	Infections (No, description) * *ABTh—antibiotic therapy, H—hospitalization*			
Patient 1 Patient 2 Patient 3	8 ABTh, 7 H; chronic cough, septic complication following gastrostomy insertion 6 ABTh, 9 H; inflammatory complications following gastrostomy insertion, slow postoperative wound healing, sepsis due to abdominal infection 7 ABTh, 5 H; frequent bacterial infections of the respiratory and GIT		3 ABTh, 0 H; predominantly viral infections, chronic cough ceased 2 ABTh, 1 H 0 ABTh, 2 H; fewer viral infections	
	G-CSF dose (µg/kg/day)			
Patient 1 Patient 2 Patient 3	1.39 0.80 1.64		Discontinued (17^th^ month) Discontinued (13^th^ month) Discontinued (17^th^ month)	
	Neutrophil burst test			
Patient 1 Patient 2 Patient 3	Abnormal, 0.11 Not performed Normal		Normal Normal Normal	
INFLAMMATION				
	Sedimentation rate median value (mm/h)			
Patient 1 Patient 2 Patient 3	120 55 120		23 18 15.5	
	Ig levels * normal values: IgG (3.86–14.7); IgA (0.29–2.56); IgM 2 (0.37–2.24) (g/L)			
Patient 1 Patient 2 Patient 3	IgG 29; IgA 3.48; IgM 1.85 IgG 13.5; IgA 1.14; IgM 0.62 IgG 25.35; IgA 6.82; IgM 2.55		IgG=19.3; IgA=2.69; IgM=1.73 IgG=19.3; IgA=2.69; IgM=1.73 IgG=13.65; IgA=3.30; IgM=2.18	
	Platelets median value			
Patient 1 Patient 2 Patient 3	450x10^9^/L 477x10^9^/L 649x10^9^/L		345x10^9^/L 418x10^9^/L 443x10^9^/L	
ANEMIA				
	Hemoglobin values (g/L), MCV values (fL)			
Patient 1 Patient 2 Patient 3	Hb: 113, MCV: 72.2 Hb: 108, MCV: 73.5 Hb: 106, MCV: 72.7		Hb: 132, MCV: 75.0 Hb: 143, MCV: 77.9 Hb: 145, MCV: 81.0	
	Iron supplementation			
Patient 1 Patient 2 Patient 3	Often required Regularly required Regularly required		Not needed Not needed Not needed	
CHRONIC INFLAMMATORY BOWEL DISEASE				
	Symptoms, histological status; * IBD—Inflammatory bowel disease			
Patient 1 Patient 2 Patient 3	Cramping abdominal pain, bloody stools Intestinal colics, poorly formed stools up to 8 times a day; no histological signs of IBD Asymptomatic, no histological signs of IBD		The pain subsided in 1 week, and stool consistency improved, mucus and blood vanished, IBD remission on histology Improved stool, occasional constipation; no histological signs of IBD Asymptomatic, no histological signs of IBD	
	Aphthous ulcers			
Patient 1 Patient 2 Patient 3	Recurrent Recurrent Constant, requires pain medications and refuses oral feeding		2 episodes after viral infection None None	
SEVERE HYPOGLYCEMIA EPISODES				
Patient 1 Patient 2 Patient 3	6 (technical problems with the enteral pump, insufficient food intake) 0 1 (skipped meal)		7 (enteral feeding pump dysfunction, physical activity, skipped meal) 0 1 (acute gastroenteritis)	
OBESITY				
	BMI (kg/m^2^)			
Patient 1 Patient 2 Patient 3	17.9 (95^th^p) 20.8 (100^th^p) 15.8 (58^th^p)		23.2 (100^th^p) 21.2 (100^th^p) 16.5 (60^th^p)	
GENERAL WELLBEING				
Patient 1 Patient 2 Patient 3	Lack of energy for physical activity Sleep problems Diminished appetite, overly sensitive		Improved, playful, energized Improved quality of sleep, improved appetite Improved, more active, improved appetite	

### 2.1 General information on empagliflozin treatment

P1, currently a 7-year-old boy, presented with gastrointestinal symptoms, frequent oral mucosa lesions, and recurrent infections. Empagliflozin was initiated at the age of 4.5 years and continued for 36 months of follow-up with an average dose of 0.38 mg/kg/day. P2, a 5-year-old boy prone to aphthous stomatitis and severe infections, commenced empagliflozin treatment at the age of 2.5 years. Over a 34-month follow-up, he received an average dose of 0.48 mg/kg/day. P3, currently an 8-year-old girl, had been suffering from constant oral mucosa lesions. Empagliflozin was introduced to P3 at the age of 6, and the course of treatment was monitored for 31 months, during which she received an average dose of 0.57 mg/kg/day. Empagliflozin therapy was administered to all three patients twice per day.

### 2.2 Infections and aphthous stomatitis

Before the introduction of empagliflozin, Patients 1–3 (P1–3) experienced recurrent bacterial infections with frequent complications, such as sepsis in P2 and chronic cough in P1. During empagliflozin treatment, the number and severity of infections have decreased, significantly reducing the need for antibiotic treatment and hospitalization. P1–3 have experienced mostly mild viral infections, and the chronic cough in P1 has ceased. The first attempt to insert a gastrostomy needed due to persistent night hypoglycemia in P1 was done prior to the empagliflozin introduction and was unsuccessful due to the severity and persistence of peristomal infection. As an alternative, a nasogastric tube was inserted, which, however, proved highly disruptive for the boy and resulted in chronic nasal discharge. In the second month of empagliflozin treatment, the gastrostomy was successfully inserted, with subsequent efficient healing of the postoperative wound.

Aphthous stomatitis was a significant issue in all three patients prior to empagliflozin use. Due to constant aphthous ulcers, P3 had impaired oral hygiene. Moreover, she required feeding via gastrostomy throughout the day, as she refused oral feeding. In the first weeks of empagliflozin treatment, a complete resolution of aphthous stomatitis was observed in P1–3. Consequently, P3 is no longer dependent on tube feeding during the day.

### 2.3 Anemia

**Figure 1:**
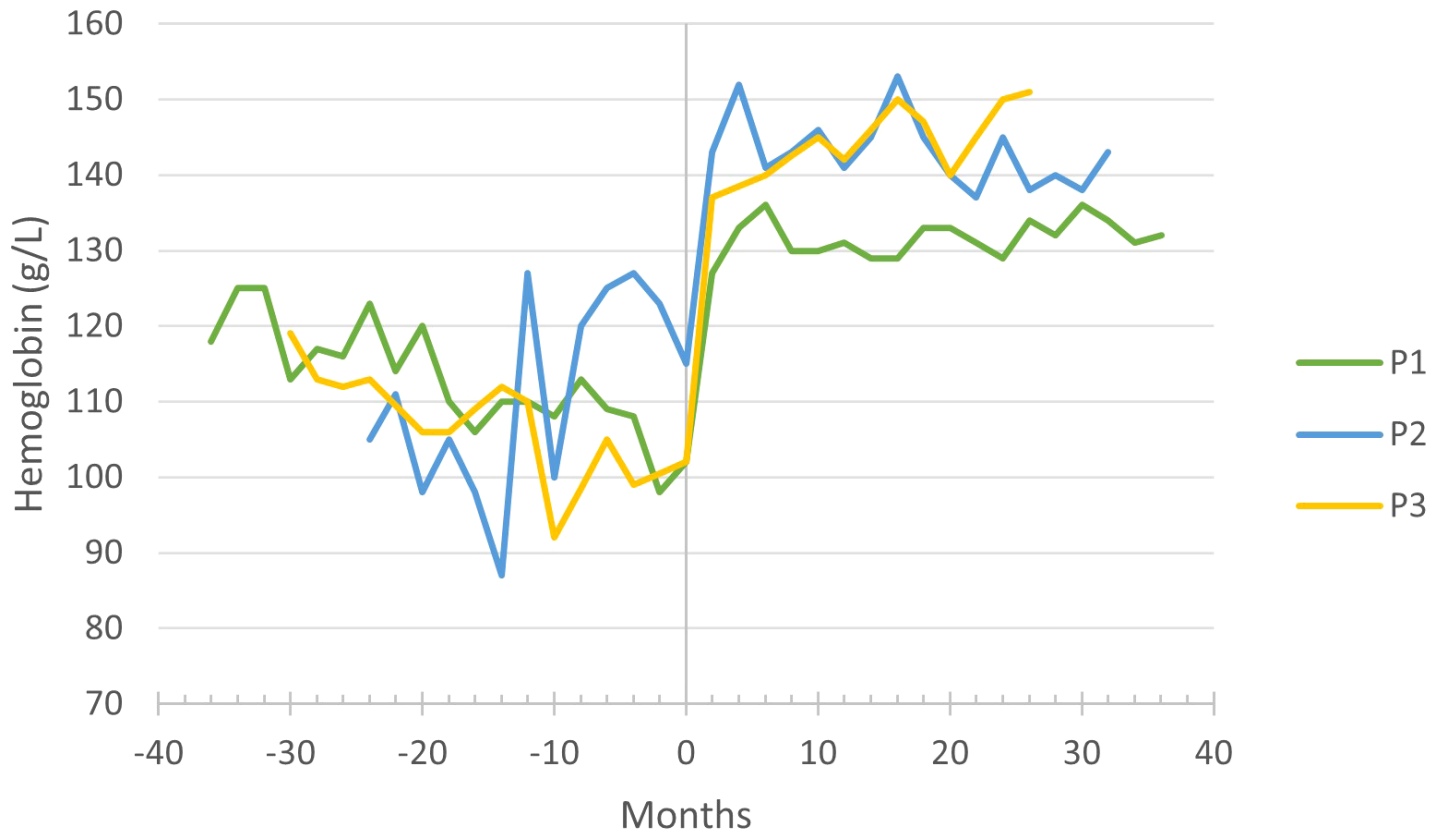
Hemoglobin levels before and during empagliflozin treatment, with month 0 representing the time of empagliflozin introduction.

P1–3 required iron supplementation due to microcytic anemia, which resolved during the first months of empagliflozin treatment. The mean corpuscular volume and hemoglobin levels have increased significantly (**Figure 1**), and the patients are no longer dependent on iron supplementation.

### 2.4 IBD and other gastrointestinal symptoms

Abdominal pain and bloody stools in P1 indicated neutropenia-related IBD, which was confirmed endoscopically and histologically at the age of 4. A few months following the diagnosis, infliximab treatment was initiated (dose: 10 mg/kg/6–12 weeks; blood concentration: median 7.1 µg/mL, range 1.2–23.6 µg/mL); however, complete relief of IBD symptoms was not achieved. With the addition of empagliflozin to the therapy, abdominal pain ceased within the first week of treatment. Stool consistency improved in the first two months of treatment, and from the fifth month onwards, mucus or blood were not visible in the stool. Fecal calprotectin, which was mildly elevated at the time of IBD diagnosis (70 mg/kg), decreased with infliximab treatment (43 mg/kg) and normalized completely following empagliflozin administration (below 16 mg/kg). Furthermore, a significant decrease in the sedimentation rate was observed (**Table 1**). Deep remission of IBD was endoscopically and histologically confirmed in the second month of empagliflozin treatment and revalidated in the 30th month, enabling the discontinuation of infliximab in the 32nd month. Even after the discontinuation of infliximab, he remained in clinical and laboratory remission. P2 and P3 have not been diagnosed with IBD.

Since birth, P2 has passed poorly formed stools multiple times a day, and the introduction of empagliflozin has normalized stool consistency and frequency.

### 2.5 General well-being and potential adverse effects

Since receiving empagliflozin, P1–3 have shown increased appetite, higher level of activity and improved overall well-being. In P2, we have observed improved sleep quality, reduced breathing pauses and saturation drops during sleep. Before the introduction of empagliflozin, P1 frequently experienced symptomatic hypoglycemias, occasionally leading to seizures. A few hypoglycemic episodes also occurred during empagliflozin treatment due to strenuous exercise or inadequate food intake; however, the frequency remained unchanged with empagliflozin administration. P3 experienced a single episode of hypoglycemia during empagliflozin treatment, which occurred due to acute gastroenteritis. Continuous glucose monitoring, employed to control blood glucose in P1–3, detected asymptomatic drops of glucose below threshold in P1 and P2 in the early morning hours prior to empagliflozin treatment; however, these asymptomatic nocturnal hypoglycemic events were significantly reduced with empagliflozin administration. P2 tolerates longer intervals between meals since treated with empagliflozin; moreover, he has successfully transitioned from continuous enteral to bolus nocturnal feeding.

**Figure 2:**
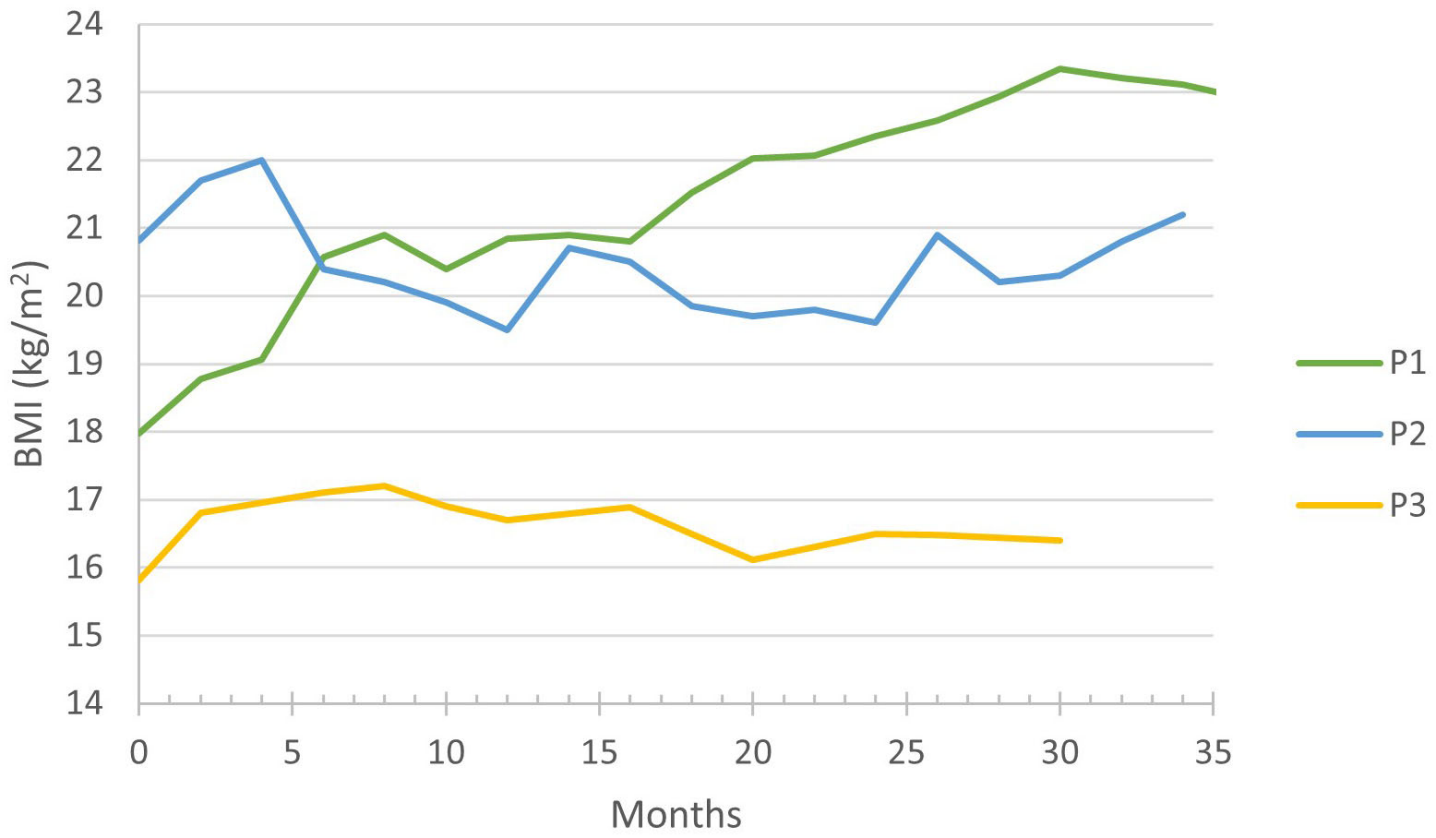
Evolution of body mass index (BMI) during empagliflozin treatment.

During the follow-up period, an increase in body mass index (BMI) was observed in P1 and P3: in P1 from 17.9 kg/m^2^ (95^th^ percentile) to 23.2 kg/m^2^ (100^th^ percentile) and in P3 from 15.8 kg/m^2^ (58^th^ percentile) to 17.2 kg/m^2^ (83^rd^ percentile), finally stabilizing at 16.5 kg/m^2^ (60^th^ percentile), (**Figure 2**). Whilst P2 has maintained a stable BMI, careful adjustments of his carbohydrate intake through the implementation of bolus nocturnal feeding were required to prevent a BMI increase.

### 2.6 Evolution and management of neutropenia

**Figure 3:**
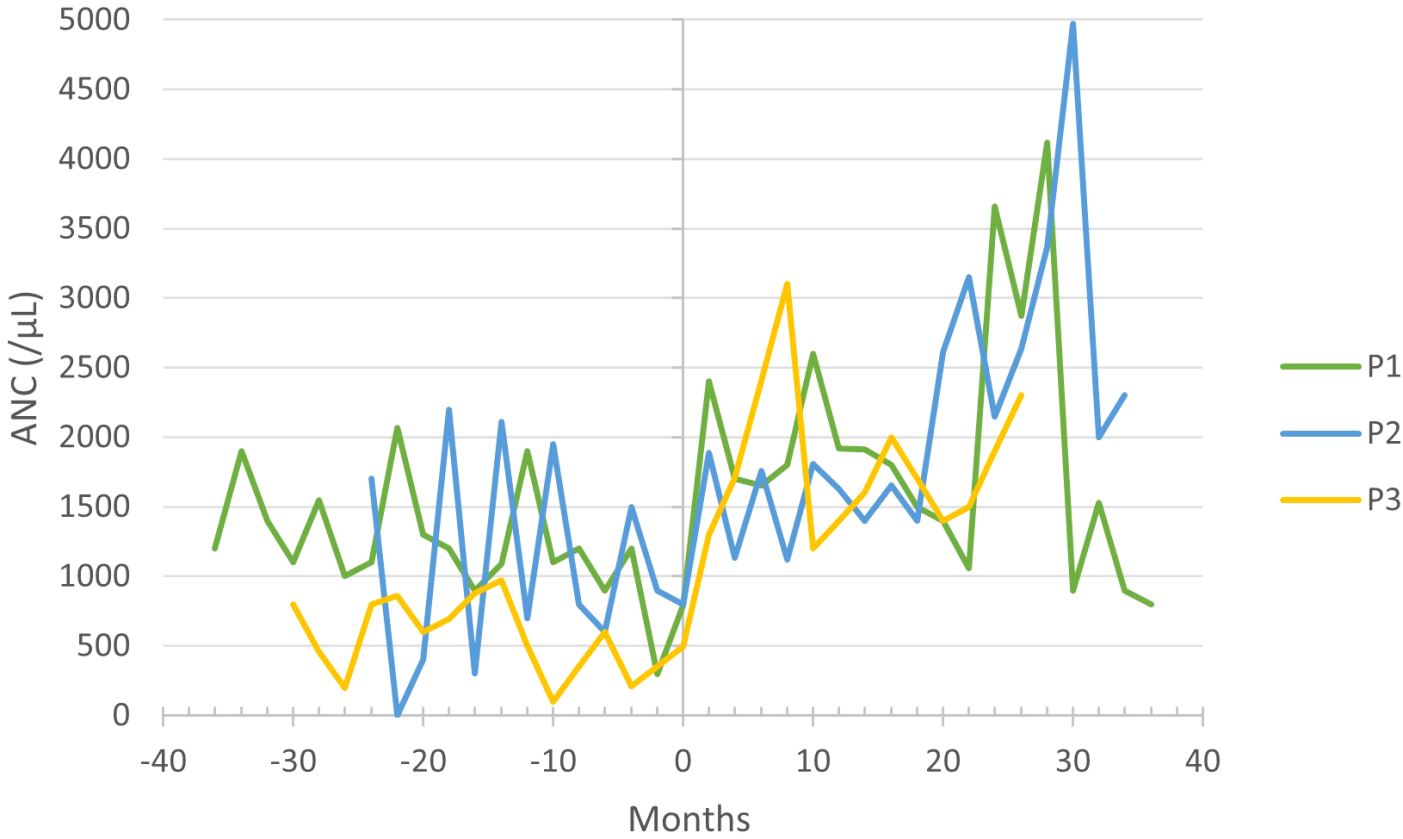
Absolute neutrophil count (ANC) before and during empagliflozin treatment, with month 0 representing the time of empagliflozin introduction.

P1–3 received G-CSF treatment before commencing with empagliflozin; however, it did not prevent neutropenia-related symptoms, and absolute neutrophil count (ANC) often remained within the neutropenic range (**Figure 3**). Upon initiating empagliflozin, P1, P2, and P3 received 1.39, 0.8 and 1.64 µg/kg/day of G-CSF, respectively. Before empagliflozin treatment, P1 experienced falls in ANC down to 300/µL, along with neutrophil dysfunction, demonstrated by nitroblue tetrazolium (NBT) test. During empagliflozin treatment, the median ANC increased, as shown in **Table 1**, and neutrophil function improved. P2 also had extensive fluctuations in ANC, but with the administration of empagliflozin, the ANC stabilized above 1100/µL. P3 presented with severe chronic cough, which resolved entirely during empagliflozin treatment. The resolution of neutropenia, coupled with favorable clinical observations, enabled the discontinuation of G-CSF in P1–3 (**Table 1**).

### 2.7 Other laboratory parameters

Elevated sedimentation rate, hypergammaglobulinemia, and thrombocytosis were detected in P1–3 prior to empagliflozin introduction. Subsequently, all patients observed a gradual decrease in immunoglobulin levels, platelets, and erythrocyte sedimentation rate during empagliflozin treatment.

In P2, who had persistent hyperuricemia, the median urate values decreased from 491 to 346 µmol/L, reflecting markedly improved metabolic control of the GSD.

## 3 Discussion

SGLT2 inhibitors are an innovative treatment option in GSD-1b patients, as they treat the cause of neutropenia by eliminating the toxic metabolite – 1,5-AG6P – from neutrophils (3). In this article, we report the effectiveness of empagliflozin in three pediatric patients with GSD-1b treated for 36, 34 and 31 months, respectively.

Neutropenia, one of the main clinical features of GSD-1b (1), was partially remedied with G-CSF in P1 and P2, but extensive fluctuations in ANC persisted (**Figure 3**). Some case studies also observed fluctuating ANC before introducing empagliflozin (6, 7, 10). In P3, severe neutropenia persisted despite treatment with G-CSF. The majority of patients included in the multicenter study also suffered due to severe or moderate neutropenia before empagliflozin treatment despite receiving G-CSF (15). The stabilization of ANC due to empagliflozin allowed us to discontinue G-CSF altogether without any complications in all patients. In contrast, two multicenter studies featuring a larger patient cohort reported G-CSF discontinuation in only about half of the cases (15, 16). Furthermore, our findings are consistent with previously published case reports, affirming that empagliflozin administration stabilizes ANC and enhances neutrophil function (6, 8–10, 14).

Improved neutrophil count and function are reflected in the significant clinical improvement in all three patients. The number and severity of infections decreased, resulting in a notable reduction in the need for antibiotic treatment and hospitalization. The gastrostomy incision site recovered almost uneventfully in P1, indicating an improved wound healing as previously reported by Grünert et al. (14) and Kaczor et al. (13). The importance of anemia resolution (**Figure 2**) under empagliflozin should be emphasized, as anemia in GSD-1b patients was quite resistant to treatment in P1–3, responding insufficiently to iron supplements. The resolution of aphthous stomatitis, reduction in both frequency and severity of infections, and alleviation of IBD symptoms collectively contribute to an enhancement in the overall well-being of the patients. Moreover, our observations reveal favorable impacts of empagliflozin on the patients’ sleep patterns and level of activity. The effectiveness of empagliflozin in alleviating neutropenia-related symptoms and improving overall well-being in GSD-1b patients is also emphasized in literature (6–18). A decrease in inflammatory markers and immunoglobulins during empagliflozin treatment could indicate an improvement in the overall immune function.

At the onset of IBD in P1, the clinical condition was partially alleviated with the introduction of infliximab. With the addition of empagliflozin to the therapy, long-term deep remission of IBD was achieved, allowing us to discontinue infliximab treatment without experiencing any exacerbations. Per our experience with GSD-1b, managing IBD is crucial for the long-term management of the disease. The role of empagliflozin in maintaining long-term remission of IBD is thus perhaps one of its most significant effects on the course of the disease. A significant proportion of GSD-1b patients treated with empagliflozin reported improved IBD symptoms (6, 7, 9–12, 14–16). Rossi et al. observed histological improvement within six months of empagliflozin treatment; however, endoscopic examination revealed persistent ulcers and strictures (7). In contrast, our patient with IBD achieved deep endoscopic and histological remission. The additional role of infliximab cannot be excluded and could indicate the need for further clinical studies.

In our patients, the frequency of hypoglycemic episodes remained unchanged compared to the period before empagliflozin was introduced. Nevertheless, some cases described in literature indicate an increased occurrence of hypoglycemia upon commencement of empagliflozin treatment (8, 16). Conversely, 50% of patients in the multicenter study reported amelioration of severe hypoglycemia under empagliflozin treatment (16). Overall, hypoglycemia is the most frequently reported adverse effect associated with empagliflozin treatment in the literature (15).

We want to draw attention to the increase in BMI in P1 and P3 (**Table 1**; **Figure 3**), which is probably due to an improved appetite and bowel health. Tallis et al. and Bidiuk et al. reported similar findings regarding weight gain (9, 18). Therefore, additional research is required to address the potential weight gain associated with the long-term use of empagliflozin.

## 4 Conclusion

This report assesses the experience with empagliflozin treatment in three pediatric patients with GSD-1b over a follow-up of three years. Clinical and laboratory observations, such as deep remission of neutropenia-related IBD, indicate that targeted metabolic treatment could improve immune function in GSD-1b patients. We observed that weight gain presents a potential issue associated with the long-term use of empagliflozin.

Multicenter follow-up studies are required to adequately evaluate the long-term influence of empagliflozin treatment in GSD-1b patients, as the disease is rare and case series reports, such as the one presented, have a limited impact. However, according to the outcomes in our long-term follow-up, the outlook of the treatment is promising., the outlook of the treatment is promising.

## Data availability statement

The original contributions presented in the study are included in the article/supplementary material. Further inquiries can be directed to the corresponding author.

## Ethics statement

The studies involving humans were approved by The National Medical Ethics Committee of the Republic of Slovenia (No. of the approval 0120-464/2023/3). The studies were conducted in accordance with the local legislation and institutional requirements. Written informed consent for participation in this study was provided by the participants’ legal guardians/next of kin. Written informed consent was obtained from the minor(s)’ legal guardian/next of kin for the publication of any potentially identifiable images or data included in this article.

## Author contributions

AK: Conceptualization, Data curation, Formal analysis, Investigation, Writing – original draft, Writing – review & editing. UG: Conceptualization, Formal analysis, Funding acquisition, Investigation, Methodology, Project administration, Resources, Supervision, Validation, Visualization, Writing – review & editing. MM: Investigation, Methodology, Validation, Writing – review & editing. MH: Investigation, Supervision, Validation, Writing – review & editing. GM: Investigation, Methodology, Supervision, Validation, Writing – review & editing. AM: Investigation, Validation, Writing – review & editing. AC: Investigation, Validation, Writing – review & editing. JS: Methodology, Project administration, Supervision, Validation, Writing – review & editing. TB: Funding acquisition, Resources, Supervision, Validation, Writing – review & editing. MZ: Conceptualization, Investigation, Project administration, Supervision, Validation, Writing – review & editing. AD: Conceptualization, Data curation, Formal analysis, Investigation, Methodology, Project administration, Supervision, Validation, Visualization, Writing – review & editing.

## References

[1] Kishnani PS Austin SL Abdenur JE Arn P Bali DS Boney A. American College of Medical Genetics and Genomics. Diagnosis and management of glycogen storage disease type I: a practice guideline of the American College of Medical Genetics and Genomics. Genet Med. (2014) 16:e1. doi: 10.1038/gim.2014.128 25356975

[2] Sim SW Weinstein DA Lee YM Jun HS. Glycogen storage disease type Ib: role of glucose-6-phosphate transporter in cell metabolism and function. FEBS Lett. (2020) 594:3–18. doi: 10.1002/1873-3468.13666 31705665

[3] Veiga-da-Cunha M Chevalier N Stephenne X Defour JP Paczia N Ferster A. Failure to eliminate a phosphorylated glucose analog leads to neutropenia in patients with G6PT and G6PC3 deficiency. Proc Natl Acad Sci U S A. (2019) 116:1241–50. doi: 10.1073/pnas.1816143116 PMC6347702 30626647

[4] Visser G Rake JP Fernandes J Labrune P Leonard JV Moses S. Neutropenia, neutrophil dysfunction, and inflammatory bowel disease in glycogen storage disease type Ib: results of the European Study on Glycogen Storage Disease type I. J Pediatr. (2000) 137:187–91. doi: 10.1067/mpd.2000.105232 10931410

[5] Diederich J Mounkoro P Tirado HA Chevalier N Van Schaftingen E Veiga-da-Cunha M. SGLT5 is the renal transporter for 1,5-anhydroglucitol, a major player in two rare forms of neutropenia. Cell Mol Life Sci CMLS. (2023) 80:259. doi: 10.1007/s00018-023-04884-8 37594549 PMC10439028

[6] Wortmann SB Van Hove JLK Derks TGJ Chevalier N Knight V Koller A. Treating neutropenia and neutrophil dysfunction in glycogen storage disease type Ib with an SGLT2 inhibitor. Blood. (2020) 136:1033–43. doi: 10.1182/blood.2019004465 PMC7530374 32294159

[7] Rossi A Miele E Fecarotta S Veiga-da-Cunha M Martinelli M Mollica C. Crohn disease-like enterocolitis remission after empagliflozin treatment in a child with glycogen storage disease type Ib: a case report. Ital J Pediatr. (2021) 47:149. doi: 10.1186/s13052-021-01100-w 34215305 PMC8254289

[8] Halligan RK Dalton RN Turner C Lewis KA Mundy HR. Understanding the role of SGLT2 inhibitors in glycogen storage disease type Ib: the experience of one UK centre. Orphanet J Rare Dis. (2022) 17:195. doi: 10.1186/s13023-022-02345-2 35549996 PMC9096769

[9] Tallis E Karsenty CL Grimes AB Karam LB Elsea SH Sutton VR. Untargeted metabolomic profiling in a patient with glycogen storage disease Ib receiving empagliflozin treatment. JIMD Rep. (2022) 63:309–15. doi: 10.1002/jmd2.12304 PMC9259396 35822097

[10] Hexner-Erlichman Z Veiga-da-Cunha M Zehavi Y Vadasz Z Sabag AD Tatour S. Favorable outcome of empagliflozin treatment in two pediatric glycogen storage disease type 1b patients. Front Pediatr. (2022) 10:1071464. doi: 10.3389/fped.2022.1071464 36507137 PMC9727171

[11] Makrilakis K Barmpagianni A Veiga-da-Cunha M. Repurposing of empagliflozin as a possible treatment for neutropenia and inflammatory bowel disease in glycogen storage disease type Ib: A case report. Cureus. (2022) 14:e27264. doi: 10.7759/cureus.27264 36039216 PMC9403211

[12] Mikami M Arai A Mizumoto H. Empagliflozin ameliorated neutropenia in a girl with glycogen storage disease Ib. Pediatr Int. (2021) 63:1394–6. doi: 10.1111/ped.14629 34378838

[13] Kaczor M Greczan M Kierus K Ehmke Vel Emczyńska-Seliga E Ciara E Piątosa B. Sodium-glucose cotransporter type 2 channel inhibitor: Breakthrough in the treatment of neutropenia in patients with glycogen storage disease type 1b? JIMD Rep. (2022) 63:199–206. doi: 10.1002/jmd2.12278 35433171 PMC8995836

[14] Grünert SC Elling R Maag B Wortmann SB Derks TGJ Hannibal L. Improved inflammatory bowel disease, wound healing and normal oxidative burst under treatment with empagliflozin in glycogen storage disease type Ib. Orphanet J Rare Dis. (2020) 15:218. doi: 10.1186/s13023-020-01503-8 32838757 PMC7446198

[15] Grünert SC Derks TGJ Adrian K Al-Thihli K Ballhausen D Bidiuk J. Efficacy and safety of empagliflozin in glycogen storage disease type Ib: Data from an international questionnaire. Genet Med. (2022) 24:1781–8. doi: 10.1016/j.gim.2022.04.001 35503103

[16] Grünert SC Venema A LaFreniere J Schneider B Contreras E Wortmann SB. Patient-reported outcomes on empagliflozin treatment in glycogen storage disease type Ib: An international questionnaire study. JIMD Rep. (2023) 64:252–8. doi: 10.1002/jmd2.12364 PMC10159866 37151361

[17] Grünert SC Rosenbaum-Fabian S Schumann A Selbitz AC Merz W Gieselmann A. Two successful pregnancies and first use of empagliflozin during pregnancy in glycogen storage disease type Ib. JIMD Rep. (2022) 63:303–8. doi: 10.1002/jmd2.12295 PMC9259388 35822091

[18] Bidiuk J Gaciong ZA Sobieraj P. The overall benefits of empagliflozin treatment in adult siblings with glycogen storage disease type Ib: one year experience. Arch Med Sci AMS. (2022) 18:1095–9. doi: 10.5114/aoms/150029 PMC9266796 35982912

